# Broadband and thin magnetic absorber with non-Foster metasurface for admittance matching

**DOI:** 10.1038/s41598-017-07323-4

**Published:** 2017-07-31

**Authors:** Jinchao Mou, Zhongxiang Shen

**Affiliations:** School of Electrical and Electronic Engineering, Nanyang Technological University, 50 Nanyang Avenue, Singapore, 639798 Singapore

## Abstract

One of the long-standing and challenging problems in microwave engineering is the realization of ultra-wideband absorption using extremely-thin structures. Magnetic material can facilitate thickness reduction for microwave absorbers but also bring inherent narrowband admittance matching conundrum originating from its frequency-dispersive permeability and high permittivity. In this paper, we propose a simple and yet effective solution based on the concept of admittance matching with non-Foster metasurface (NFMS). Building on this concept, an ultra-wideband and extremely-thin magnetic absorber is achieved, with a simple structure consisting of a conductor-backed magnetic sheet (CMBS) coated by a NFMS. The NFMS with negatively inductive susceptance can properly cancel its positively frequency-dispersive counterpart from the CMBS so that constructive interference near the absorber can be obtained over a wide frequency band. Furthermore, the NFMS will compensate the surface conductance required for maximum incident power dissipation. As an example, we demonstrate an absorber with one-frequency decade bandwidth and a thickness of only 1/255 wavelength at the lowest operation frequency. The proposed concept enables versatile admittance matching techniques using a single-layered and has the potential to be used in the development of interesting low-profile and broadband microwave devices.

## Introduction

Achieving wideband absorption while maintaining a low profile has been a major problem in the field of electromagnetic (EM) wave control and manipulation since early days^1–4^. Even today, many applications require simple and thin absorbers exhibiting the widest absorption bandwidth, such as radar cross section reduction, EM compatibility, and wireless communications^5–9^. Unfortunately, bandwidth enhancement and thickness reduction is a pair of fundamental contradictory propositions for absorber designs, which has been mathematically illustrated by the Rozanov theory^10^. For example, Jaumann layers^11^ can successfully broaden the absorption bandwidth but suffer from much higher profiles compared to the single-layer Salisbury screens^12^. An extremely thin absorber can be achieved based on metamaterial, but has very narrow bandwidth^13–15^. One effective approach is to coat frequency selective surface (FSS)^16–23^ or multi-layered metasurface^24–27^ on a nonmagnetic substrate to widen the bandwidth while reducing thickness. Several designs based the coating method can approach to but never be able to break the ultimate thickness-to-bandwidth ratio defined by the Rozanov limit^18^. This may be a serious problem for long-wavelength band applications. Consider a simple case to clarify this point of view. Suppose an absorber with nonmagnetic substrate shows good absorption (>90%) from 100 to 1000 MHz, the minimum thickness according to the Rozanov theory^10^ is 300 mm, which may be too thick to be practically useful.

Magnetic material can facilitate further thickness reduction compared with nonmagnetic solutions. Although a guideline is provided^28^ for realizing broadband absorber using very thin magnetic substrate, it appears that realistic materials do not always exhibit such idea constitutive parameters due to the fundamental restrictions imposed by the Kramers-Kronig relationship^29, 30^ and particle resonances^31^. This implies that it is fundamentally impossible to obtain broadband absorption with homogeneous, single-layer magnetic materials^29, 32, 33^, which usually shows narrowband or out-of-the-desired-band absorption. Analogous to nonmagnetic designs, FSS or metasurface^34–38^ can coat the magnetic sheet for bandwidth enhancement. However, the increased bandwidth is confined and sometimes much narrower compared with its nonmagnetic analogues. The primary cause lies in the highly frequency-dispersive input admittance of the conductor-backed magnetic sheet *Y*_*S*_. It has been discussed that passive coating can only manipulate the admittance of a passive EM structure within inherently limited bandwidth, both of which have to follow Foster dispersion^39^. The other important concern is that passively screened magnetic absorbers cannot violate the Rozanov limit^10^. Specifically, let’s continue discussing the previous example by replacing the nonmagnetic substrate with magnetic materials. For simplification, we assume the magnetic material possesses constant relative permeability *μ*_*r*_ = 5 and relative permittivity *ε*_*r*_ = 16, with both the dielectric and magnetic loss ignored. The theoretical thickness of the magnetic-based absorber with bandwidth from 100 to 1000 MHz should be at least 60 mm. It is much thinner than its nonmagnetic counterpart but still too thick for many applications, such as serving on a stealth coating for airplanes.

From the discussions, it follows that magnetic materials may facilitate absorber’s thickness reduction, whereas expanding the bandwidth of a thin magnetic-based absorber is a challenge. The main difficulty, from the admittance point of view, is how to tailor *Y*_*S*_ to the free-space admittance *Y*_*0*_ throughout a broad frequency band of interest? Active scheme may provide a possible solution^40–42^. In our previous investigation^43^, we have proposed the concept of non-Foster absorbers, where an admittance matching layer with non-Foster elements is designed to counteract the susceptance of the conductor-backed substrate, based on thin lossless dielectrics. Can non-Foster matching layer promise essential application opportunities in design of magnetic absorbers, where *Y*_*S*_ is plural and frequency-dispersive?

In this paper, we investigate the admittance matching feasibility by using a single-layered non-Foster metasurface (NFMS) to properly manipulate *Y*_*S*_ of a conductor-backed magnetic sheet (CBMS) so that a good match to *Y*_*0*_ is achieved. Based on this concept, an ultra-wideband and extremely-thin absorber is demonstrated, which is a significant improvement compared with our previous design. Furthermore, this study broadens the notion of non-Foster absorbers into a more general case, where the admittance to be matched is not only positively inductive but also plural and highly frequency-dispersive. Therefore, the NFMS should be characterized by two features, including negatively inductive susceptance with equally dispersion degree as Im(*Y*_*S*_) for constructive interference, and surface conductance to adequately compensate Re(*Y*_*S*_) for maximum incident power dissipation. We demonstrate theoretically and experimentally an absorber with a total thickness of only 7.35 mm and an absorption frequency band from 160 to 1000 MHz. This work opens up the possibility for a more general and flexible admittance matching approach, facilitating not only absorber designs but also other low-profile microwave devices.

## Results

### Configuration and Design Concept

**Figure 1:**
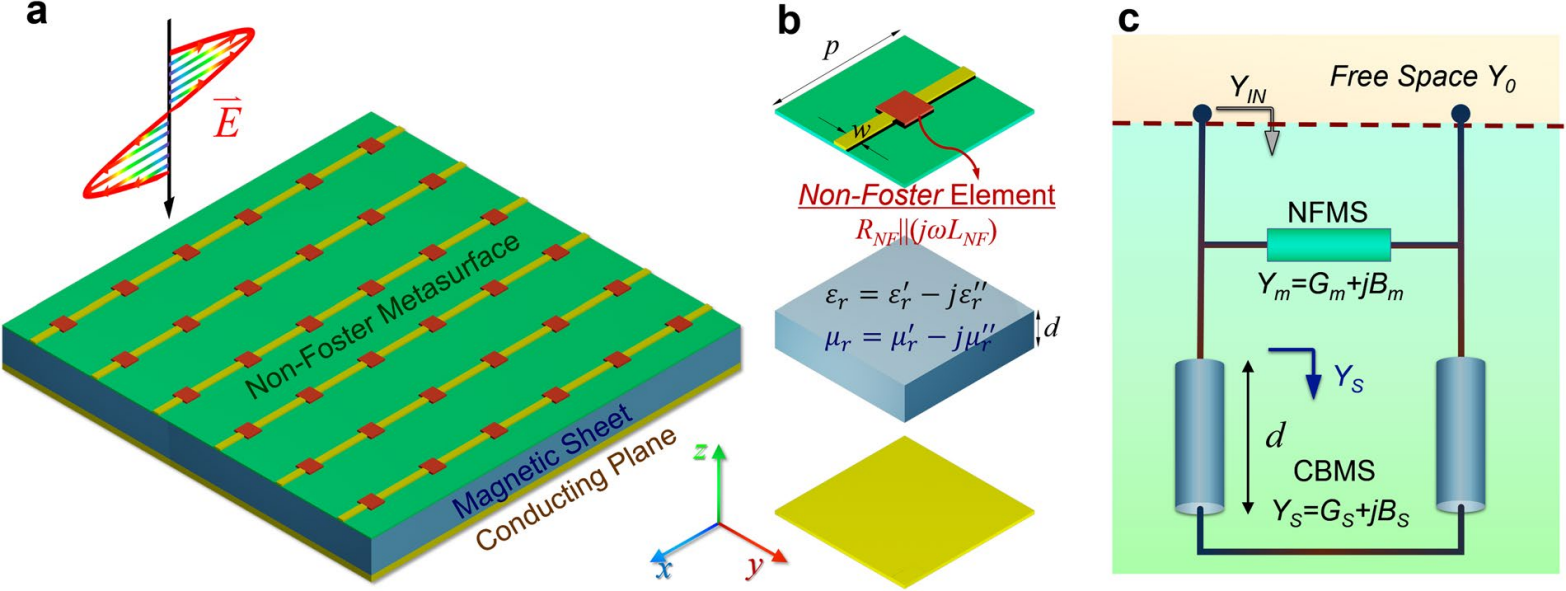
Configuration and design concept of the proposed NFMS magnetic absorber. (**a**) Conceptual structure of the NFMS magnetic absorber. It is a two-dimensional infinite periodic structure and only 6 × 6 unit cells are demonstrated. (**b**) Expanded view of the unit cell with each layer separated. The non-Foster element is an active circuit in practical, and can be represented by a lumped device due to its electrically-small footprint. (**c**) Transmission line model of the NFMS magnetic absorber based on microwave transmission line theory. *Y*_*0*_ is the free-space admittance, and *Y*_*IN*_ is the input admittance of the absorber at the interface between the free space and absorber.

Figure 1a illustrates the conceptual configuration of the NFMS magnetic absorber proposed in this paper, containing three parts in stack. A highly conductive ground plane is located at the bottom, which prevents incident waves from propagating and thus ensures a zero transmissivity. A thin magnetic sheet with the relative permittivity *ε*_*r*_ = *ε*_*r*_ *′* − *jε*_*r*_ *″*, relative permeability *μ*_*r*_ = *μ*_*r*_ *′* − *jμ*_*r*_ *″* and thickness *d* (*d* ≪ *λ*), serves as the substrate. The non-Foster metasurface with unit cell periodicity *p* is coated on the magnetic sheet. As depicted in Fig. 1(b), each unit cell of the metasurface consists of a non-resonating but polarization-sensitive strip integrated with a non-Foster element, facilitating single-polarized absorption and convenient polarization-independent expansion. Since *p* is much smaller than the operation wavelength, the NFMS can be characterized by an averaged surface admittance *Y*_*m*_ = *G*_*m*_ + *jB*_*m*_ under transverse-magnetic (TM) plane wave illumination.

We can illustrate the proposed NFMS magnetic absorber from the admittance matching point of view, with the aid of an equivalent model based on microwave transmission line theory. As depicted in Fig. 1c, the CBMS is modeled as a short-circuited transmission with length *d*, with *Y*_*m*_ in shunt connection due to the stacked configuration. According to microwave transmission line theory, the impedance of a short-circuited lossy transmission line (length < λ/4) is inductive. As a result, the admittance of the grounded magnetic substrate *Y*_*S*_ is characterized by a shunt connection of a conductance *G*_*S*_ and susceptance *B*_*S*_.

The absorptivity of the absorber may be expressed by

1 $$A=1-R=1-{|\frac{{Y}_{0}-{Y}_{IN}}{{Y}_{0}+{Y}_{IN}}|}^{2}$$

where *Y*_*IN*_ = *G*_*IN*_ + *jB*_*IN*_ *= Y*_*m*_ + *Y*_*S*_. The input admittance *Y*_*S*_ of the CBMS can be formulated by

2 $${Y}_{S}={G}_{S}+j{B}_{S}={Y}_{0}\sqrt{\frac{{\varepsilon }_{r}}{{\mu }_{r}}}\,\coth [j\frac{\omega }{{c}_{0}}\sqrt{{\varepsilon }_{r}{\mu }_{r}}d]$$

Intuitively from equation (1), there should be *Y*_*IN*_ = *Y*_*0*_ if we want to achieve perfect absorption (***A*** = **1**) over all frequencies. As a result, we can obtain the required *Y*_*m*_ of the NFMS as follows,

3 $${G}_{m}={Y}_{0}-\mathrm{Re}({Y}_{S})\quad {\rm{and}}\quad {B}_{m}=-{\rm{Im}}({Y}_{S})$$

This is the admittance matching condition for a magnetic absorber with NFMS. The NFMS will present negatively inductive *B*_*m*_ to counteract the positively inductive *B*_*S*_, so that constructive interference can be formed. In addition, the NFMS will provide surface conductance *G*_*m*_ to compensate *G*_*S*_ for maximum power dissipation. These two actions facilitate the admittance tailoring of *Y*_*S*_ to *Y*_*0*_.

Since *Y*_*m*_ is closely associated with *Y*_*S*_, we first focus on *Y*_*S*_. *Y*_*S*_ can be expanded into a Taylor series

4 $${Y}_{S}={Y}_{0}\sqrt{{\varepsilon }_{r}/{\mu }_{r}}[\frac{{c}_{0}}{j\omega \sqrt{{\varepsilon }_{r}{\mu }_{r}}d}+\sum _{n=1}^{\infty }\frac{{2}^{2n}{B}_{2n}{(j\sqrt{{\varepsilon }_{r}{\mu }_{r}}d/{c}_{0})}^{2n-1}{\omega }^{2n-1}}{(2n)!}]$$

where B2n is Bernoulli number. Equation (4) indicates that *Y*_*S*_ is a complicated function of frequency *ω*. If a lossless and thin dielectric material (*ε*_*r*_ = *ε*_*r*_ *′*, *μ*_*r*_ = 1) is used as the substrate, as the case in our previous investigation, equation (4) can be simplified into a purely inductive susceptance *Y*_*S*_ ≈ (*jμ*_*0*_ *ωd*)^−1^. This is equivalent to a nondispersive inductance *L*_*S*_ = *μ*_*0*_ *d*. Therefore, equation (3) simplifies into Re(*Y*_*m*_) = *Y*_*0*_ and Im(*Y*_*m*_) = −(*jμ*_*0*_ *ωd*)^−1^, meaning that the NFMS can tailor *Y*_*S*_ to *Y*_*0*_ with a constant surface conductance *Y*_*0*_ and negative inductance (−*μ*_*0*_ *d*). However, for the magnetic case, both *ε*_*r*_ and *μ*_*r*_ are complex number, and more importantly, *μ*_*r*_ is usually frequency-dispersive. Therefore, *Y*_*S*_ is plural and associated with higher power of *ω*, leading to high frequency dispersion degree. To achieve the matching condition formulated in equation (3), the NFMS has to possess the following two features. First, the NFMS should provide negatively inductive *B*_*m*_ with the same dispersion degree as *B*_*S*_ within the frequencies of interest. Second, the NFMS should afford frequency-dispersive *G*_*m*_, which may be negative when *G*_*S*_ > *Y*_*0*_ (This will be illustrated with the aid of full-wave EM simulations in the supplementary material).

**Figure 2:**
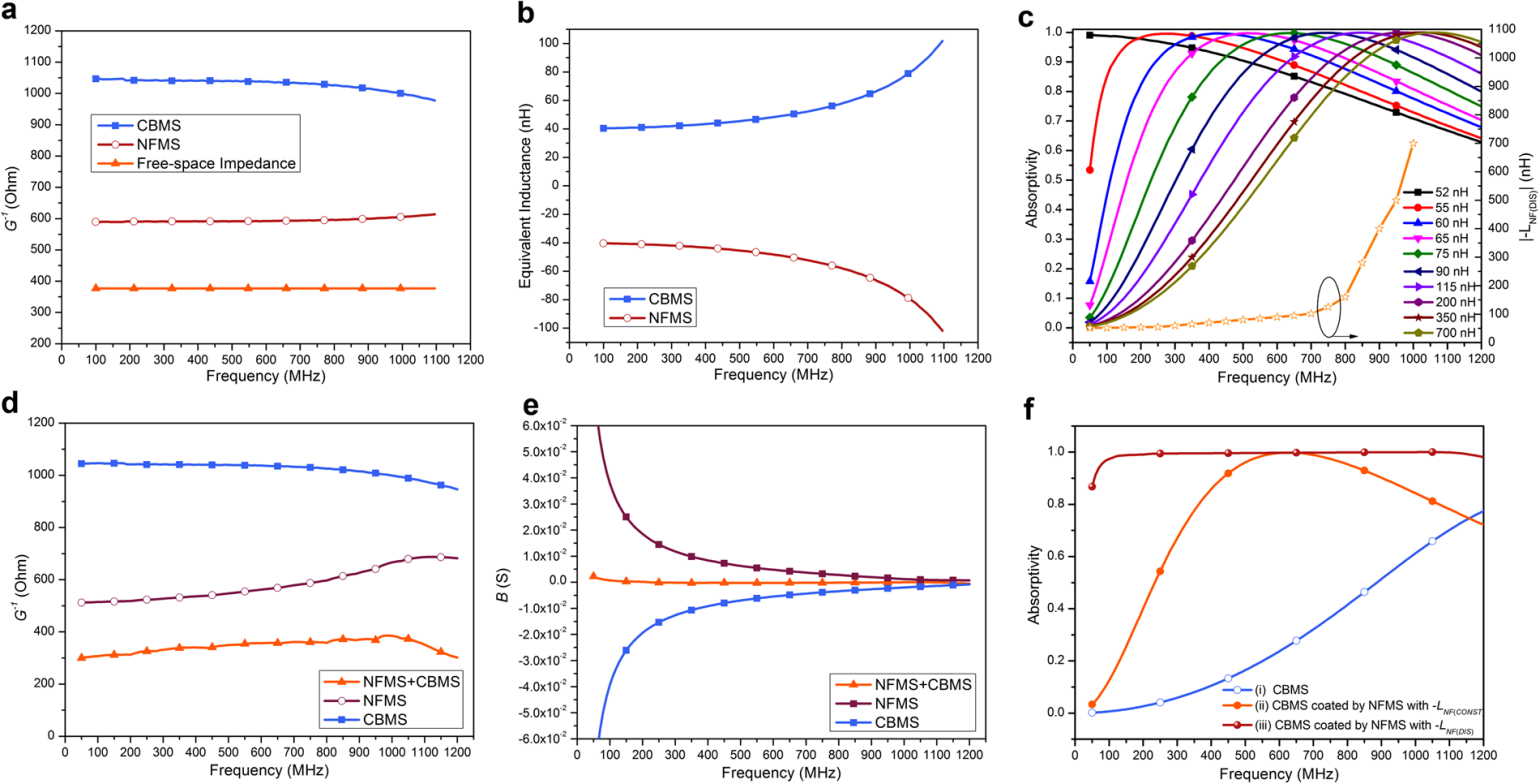
Theoretical calculation and full-wave EM simulation. (**a**,**b**) Calculated G and L of the CBMS and NFMS, according to equations (2) and (3). (**c**) Full-wave simulated absorptivity of the NFMS with different [−L_NF(CONST)_] and the translated frequency-dispersive [−L_NF(DIS)_] for perfect absorption (right y-axis). (**d**,**e**) Full-wave simulated conductance *G* and susceptance *B* of the CBMS, NFMS and NFMS magnetic absorber, based on the non-Foster element with *R*_*NF*_||[−*L*_*NF*(*DIS*)_]. (**f**) Full-wave simulated absorptivity of the following three cases: 1. CBMS only, 2. CBMS coated by NFMS with constant *R*_*NF*_||[−L_NF(CONST)_] = −75 nH, and 3. CBMS coated by NFMS with optimal dispersive *R*_*NF*_||[−L_NF(DIS)_].

Next, let’s consider a simple case without the loss of generality to illustrate the above discussions. As discussed previously, we still take the typical frequency range from 100 to 1000 MHz as the desired absorption band. A commercial silicone loaded magnetic sheet from ARC Inc., with a thickness of *d* = 6.35 mm (i.e., 1/472 *λ*_*L*_ at the lowest frequency), is used as the substrate, where its frequency-dependent *ε*_*r*_ and *μ*_*r*_, are graphically summarized in Fig. S1. Its input admittance *Y*_*S*_ is obtained according to equation (2) and plotted in Fig. 2(a) and (b), in forms of (1/*G*_*S*_)||*L*_*S*_. (1/*G*_*S*_) is larger than 1/*Y*_*0*_ = 377 Ohm with a little dispersion around 800 Ohm, whereas *L*_*S*_ shows high dispersion that increases monotonically from 40 to 80 nH within the frequency band of interest. According to equation (3), we should design a NFMS with *G*_*m*_ and *L*_*m*_ plotted in Fig. 2(a) and (b) respectively, in order to facilitate *Y*_*IN*_ = *Y*_*0*_ and further *A* = 1.

### Full-Wave EM Simulations

Following the above analysis, we design and verify the NFMS magnetic absorber with the aid of full-wave EM simulation, where the unit cell shown in Fig. 1(b) is used. The transverse dimensions of the unit cell are determined to be 20 mm × 20 mm, i.e., 1/150 *λ*_*L*_ × 1/150 *λ*_*L*_, in order to avoid higher-order current modes on the strip and maintain good matching state under oblique incident conditions. A narrow and straight strip with width *W* = 1 mm is printed on a thin dielectric substrate FR4 with *ε*_*r*_ = 4.4. The metallic strip orients along *x*-axis reacting to TM-polarized incident waves. The non-Foster element integrated at the center of the strip is an active amplifier circuit with feedback loops. In this verification, we model the non-Foster element by a lumped device with *R*_*NF*_||(−*L*_*NF*_) due to its electrically-small footprint. Its physical implementation will be discussed later with experimental demonstration. It may be mentioned that the passive strip will also introduce positive inductance *L*_*STRIP*_, which will be taken into account in the simulation.

To obtain the optimal *Y*_*m*_, we take the following two steps. First, assuming (−*L*_*NF*_) is a constant and denoted as (−*L*_*NF*(*CONST*)_), we can get a group of absorptivity of the absorber with different (−*L*_*NF*(*CONST*)_), as plotted in Fig. 2(c). Second, we translate the tuning behavior of the absorber for several discrete values of (−*L*_*NF*(*CONST*)_) into dispersive (−*L*_*NF*(*DIS*)_), which is graphically summarized on the right *y*-axis in Fig. 2(c). |−*L*_*NF*(*DIS*)_| increases monotonically from 52 to 700 nH from100 to 1000 MHz. It is reasonable that |−*L*_*NF*(*DIS*)_| is more dispersive than *L*_*S*_ due to *L*_*STRIP*_. During the above procedures, *R*_*NF*_ keeps to be a constant of 800 Ohm. We plot the simulated *Y*_*m*_, *Y*_*S*_ in Fig. 2(d) and (e), facilitating *1/G*_*IN*_ ≈ 377 Ohm and *B*_*IN*_ ≈ 0. Figure 2(f) plots the simulated absorptivity of the NFMS magnetic absorber with dispersive (−*L*_*NF*(*DIS*)_), exhibiting perfect absorption throughout the frequency band of interest. For comparison, the absorptivity of two cases are also plotted, including (i) CBMS only and (ii) CBMS coated by NFMS with constant (−*L*_*NF*(*CONST*)_) = −75 nH. The first case shows low absorption (<70%) within the frequency of interest, and the second case shows good absorption (>90%) only from 440 to 900 MHz.

### Physical Insight

**Figure 3:**
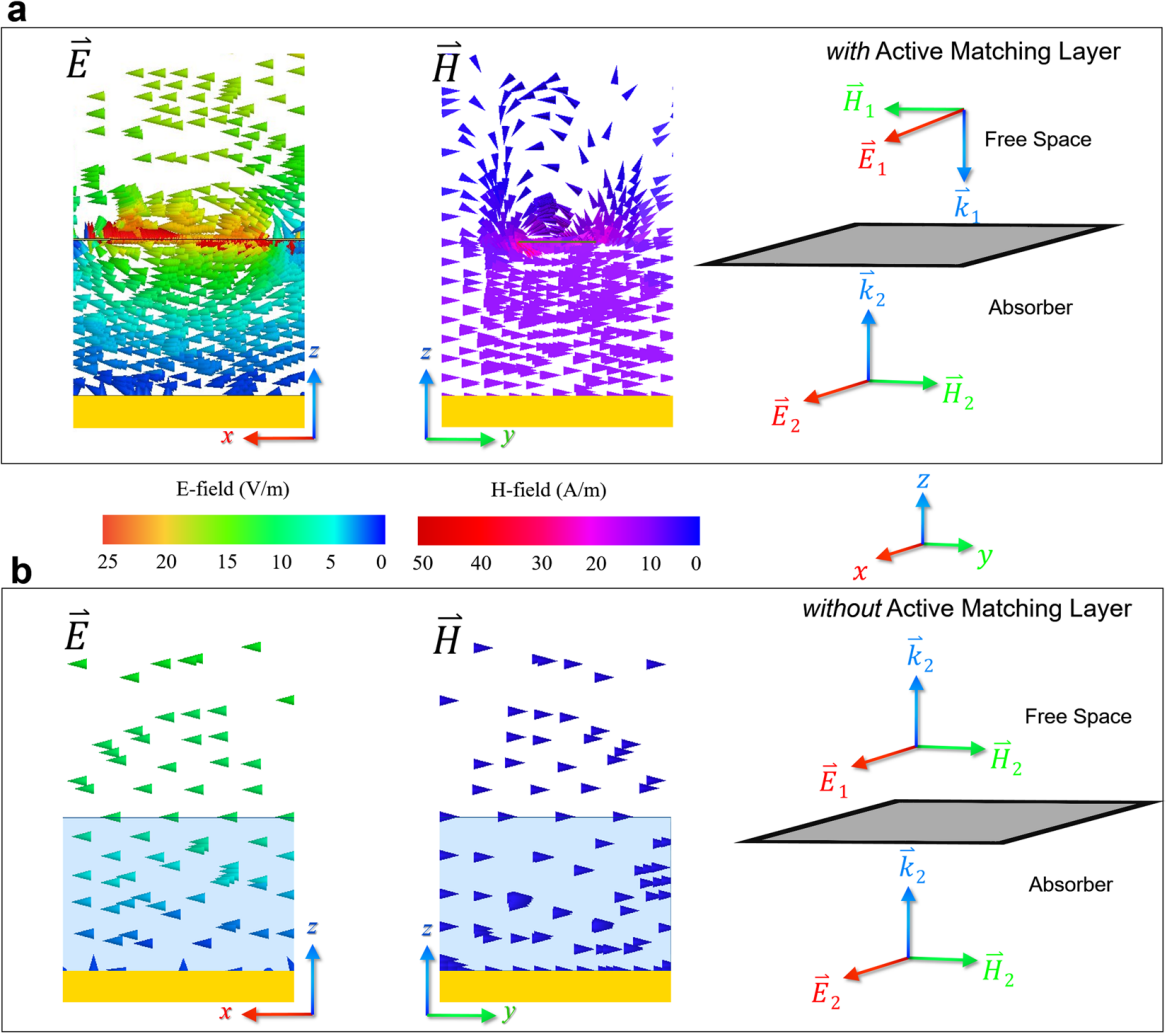
Physical Insight based on EM-field distributions. (**a**) NFMS magnetic absorber. (**b**) CBMS without coatings. E-field only distribute in *xoz*-plane and H-field only distribute in *yoz*-plane.

In order to gain a physical insight into its operation mechanism, Fig. 3(a) illustrates EM field distributions near the NFMS absorber, illuminated by a TM plane wave. Current induced on the strip will be re-phased by the non-Foster element and then then re-radiate to constructively interfere with the incident waves, resulting in maximum electrical field distribution at the interface between the free space and absorber. The concentrated power will be dissipated by the conductance both in the non-Foster element and magnetic sheet. According to the electrical- and magnetic-field vectors illustrated in Fig. 3(a), the wave vector *k* only has a (−*z*)-component above the surface of the absorber, implying low reflection and good absorption. For comparison, the field distributions near the CBMS without loading are depicted in Fig. 3(b). It is seen that the electrical field vanishes in the substrate due to the destructive interference caused by the conducting plane. In this case, lossy elements locating there won’t dissipate the incident power efficiently, leading to high reflection and weak absorption.

### Practical Implementation

**Figure 4:**
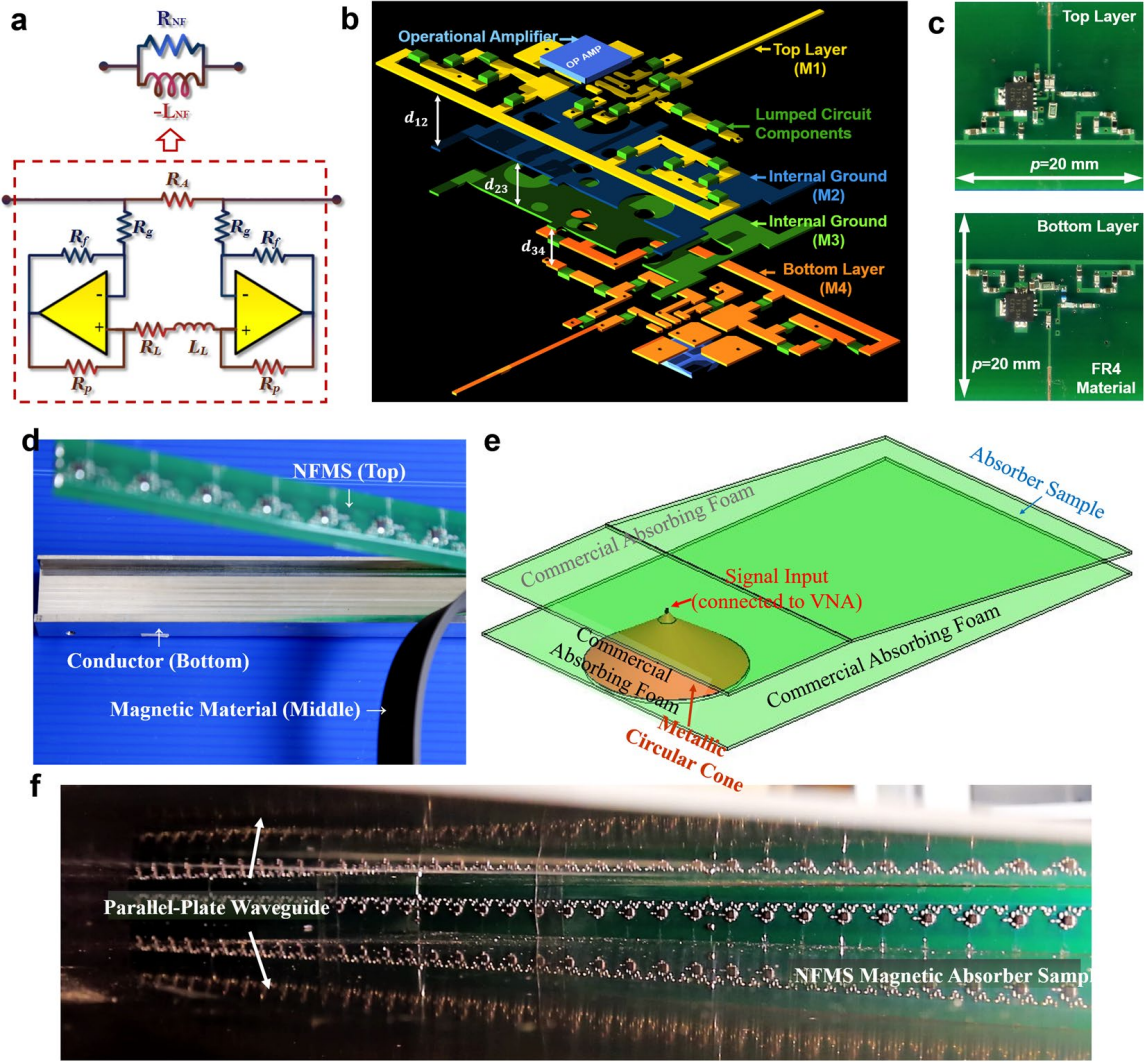
Physical Implementation of NFMS magnetic absorber. (**a**) Topology of the non-Foster element for performing as the theoretically optimal *Y*_*m*_ shown in Fig. 2(d) and (e). (**b**) Conceptual configuration of the physically implemented NFMS using a multi-layers PCB, where d_12_ = 0.4 mm, *d*_*23*_ = 0.2 mm, and *d*_*34*_ = 0.4 mm. (**c**) Top and bottom views of the fabricated NFMS. (**d**) Non-Foster absorber sample, including backed-conductor, magnetic sheet and NFMS. (**e**) 3D view of the measurement setup. (**f**) Fabricated NFMS magnetic absorber sample placed within a compact test setup based on parallel-plate waveguide.

To fit the optimal *Y*_*m*_ presented in Fig. 2(b) and (c), we design a suitable topology of the non-Foster element, as shown in Fig. 4(a). It consists of a resistor *R*_*A*_ in shunt connection with a floating negative impedance converter (FNIC). The FNIC contains two operational amplifiers (OP-AMPs) with positive and negative feedbacks so that the current can be properly re-phased to generate the desired negatively inductive susceptance. *R*_*A*_ mainly contributes to *R*_*NF*_ and the FNIC is responsible for providing (−*L*_*NF*(*DIS*)_). For compact footprint, we realize the circuit based on a 4-layers printed circuit board with metallic through-holes, as shown in Fig. 4(b). It can be treated as a single-layered surface because its total thickness is only 1/3000 λ_L_. Co-simulation and co-optimization are performed for achieving absorption bandwidth from 100 to 1000 MHz, and the realistic NFMS is shown in Fig. 4(c). The stacking sequence of the absorbing sample is illustrated in Fig. 4(d). By stacking the NFMS on the CBMS, an absorber sample including 50 cells in one row is fabricated.

**Figure 5:**
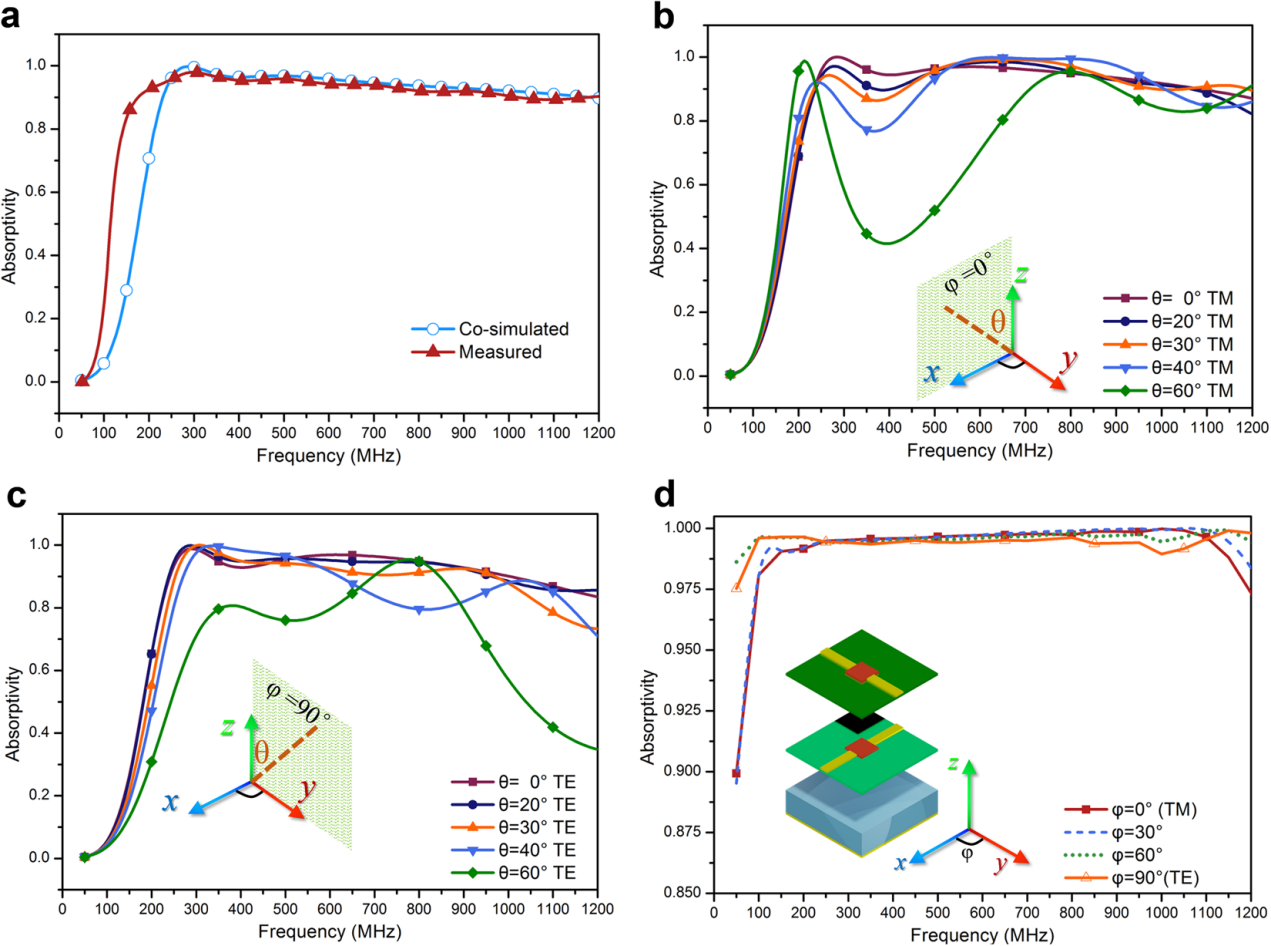
Performance of the NFMS magnetic absorber. (**a**) Measured absorptivity based on the parallel-plate waveguide based method, and simulated absorptivity based on the co-simulation method, where the layout shown in Fig. 4(b) is taken into simulation. (**b**) Simulated absorptivity under TM illumination with different oblique angles θ. (**c**) Simulated absorptivity under TE illumination with different oblique angles θ. Oblique incident results are obtained based on the same simulation approach mentioned above. (**d**) Conceptual configuration of the frequency-independent absorber’s unit cell, and its simulated absorptivity under normally incident wave with different polarization angles φ. The simulation is carried on the method used for Fig. 2(f).

A compact test fixture based on parallel-plate waveguide is shown in Fig. 4(e). The absorbing sample is placed at the end of the fixture, as illustrated in Fig. 4(f). It should be highlighted that the one-row sample is sufficient for experimental characterization according to EM image theory. The total thickness of the absorber including the NFMS and magnetic sheet is only 7.35 mm, corresponding to 1/255 λ_L_ at the measured lowest operation frequency 160 MHz. The measured absorptivity depicted in Fig. 5(a) exhibits a good absorption (>90%) from 160 to 1000 MHz, agreeing well with the full-wave EM based co-simulated one.

Figure 5(b) and (c) shows its co-simulated absorptivity under TM and TE obliquely incident illumination, revealing good absorption up to 30 degrees. It should be stressed that although the structure shown in Fig. 1(a) can only operate under single polarization, it can be conveniently extended into a polarization-independent absorber by staking the other NFMS with orthogonal polarization. Figure 5(d) illustrates the conceptual configuration of a polarization-independent NFMS absorber and its simulated absorptivity under normally incident wave with different polarization angles.

## Discussion

We have introduced a novel design for ultra-wideband and extremely-thin EM absorbers. To that end, we harness the concept of admittance matching to devise a NFMS with surface conductance and negatively inductive susceptance. The NFMS allows us to tailor the highly-dispersive input admittance of the conductor-backed thin substrate close to the free-space admittance, thus overcoming the fundamental limitation on simultaneously broadening the bandwidth and reducing the thickness for a microwave absorber.

It should be emphasized that the design concept formulated and demonstrated herein provide a general solution of broadband and thin absorber design. Once the admittance of a planar EM structure to be matched is characterized, the rest of the procedure is straightforward. The NFMS, combined with the efficient co-simulation and co-optimization approach, allows the development of desired surface admittance for susceptance cancellation and conductance compensation. In addition, considering the recent demonstrations of metasurfaces in many applications, the proposed methodology could be applied to design other wideband and low-profile microwave devices.

## Methods

### Unit Cell modeling

The unit cell of the NFMS magnetic absorber depicted in Fig. 1(b) was defined in full-wave EM simulator, ANSYS High Frequency Structure Simulation (HFSS), with a perfect electrical conductor plane (gold plane in Fig. 1), a 6.35-mm-thick silicone loaded magnetic sheet (blue box in Fig. 1) and a 1-mm-thick FR4 laminate (green box in Fig. 1). The frequency-dependent permittivity and permeability of the magnetic material, as provided to us by ARC Corporation, have been imported into the model. The non-Foster element is modeled by a lumped RLC boundary with *R*_*NF*_||(−*L*_*NF*_). Periodic boundary conditions are employed along the *x*- and *y*-directions to characterize the infinitely large structure. Floquet port with TM polarization is set for exciting a plane-wave illumination polarized in the *x*-direction. All the frequency-dependent data, such as *ε*_*r*_ = *ε*_*r*_ *′* − *jε*_*r*_ *″*, *μ*_*r*_ = *μ*_*r*_ *′*−*jμ*_*r*_ *″*, −*L*_*NF*(*DIS*)_ can be indexed with a custom piecewise function *pwlx*(*A*, freq) in HFSS.

### Co-simulation and Co-optimization method

The NFMS absorber can be divided into a passive part and an active part from the circuit network point of view. The passive part includes the passive strip, magnetic sheet and conducting plane, whereas the active part is the non-Foster element. The passive structure is simulated in HFSS, with its *S*-parameters written into a *Touchstone* file and then imported into ADS. The active part is simulated in Keysight Advanced Design System (ADS), based on the momentum 3D planar EM simulator, which can simulate complex EM effects in the non-Foster circuit, such as substrate effect and multiple dielectric layers with metallic vias. With *S*-parameters in *Touchstone* file and layout-based non-Foster circuit, the absorptivity of the NFMS absorber can be calculated and optimized in ADS’s schematic simulator. We perform co-optimization with the goal of achieving the widest absorption bandwidth throughout the frequency band from 100 to 1000 MHz and optimization variables of L_L_, R_L_, R_P_ and R_A_ in Fig. 4(a).

### NFMS and Absorber Sample Realization

For a compact design, the layout of the non-Foster circuit is realized using a 4-layers standard multi-layer printed circuit board (PCB). RF traces and DC biasing networks are arranged on the top and bottom layers, which are interconnected by metallic through-holes. The two internal layers serve as internal ground reference for circuit. As in the full-wave simulations, the fabricated NFMS consists of two 0.4-mm-thick FR4 laminates. The copper traces’ geometry for RF path and DC biasing networks is exported to standard *.*grbr* files, later used by PCM Pte. Ltd. to accordingly etch the electrodeposited 35-um-thick copper foils covering the laminates. The etched laminates are then bonded using 0.2-mm-thick prepreg-2116 bondply, forming the desirable four-layer circuit layout. Components, including operational amplifiers together with resistors, capacitors and inductors, are mounted onto PCB using surface-mount technology based on reflow-soldering.

### Microwave measurements

The fabricated absorber sample is measured within a parallel-plate waveguide testing setup that consists of a pair of parallel metallic plates, a metallic circular cone and a coaxial connector^18^. The transverse electromagnetic (TEM) wave with vertical polarization will be excited in the waveguide and propagates toward the absorber sample placed at the end of the parallel-plate waveguide. Commercial polyurethane foam absorbers, Eccosorb’s AN-79, are filled around the edges of the setup for reducing multiple reflections in the limited space. According to EM image theory, the sample inside the parallel-plate waveguide can be imaged electrically and treated as an infinite array. The test process is listed as follows. 1) The parallel-plate waveguide is surrounded by commercial polyurethane foam absorber, which imitates the free space or the environment in an anechoic chamber. The measured reflection coefficient is recorded as the background signal Γ_RAD_. 2) The foam absorber at the one side of the parallel-plate waveguide is then removed and replaced with a metallic plate with the same length and width as those of the absorber under test. The measured reflection coefficient Γ_PEC_ is again recorded as the signal completely reflected by the metallic plate. 3) The metallic plate is then replaced by the non-Foster absorber sample and the resultant signal is recorded as Γ_ABR_. The reflection of the sample under test can be obtained by |Γ| = [ ^−1^(Γ_DUT_) −  ^−1^(Γ_RAD_)]/[ ^−1^(Γ_PEC_) −  ^−1^(Γ_RAD_)], where  and  ^−1^ are the Fourier operator and inverse-Fourier operator, respectively. Γ_RAD_, Γ_PEC_, and Γ_DUT_ are measured reflection coefficients in the frequency domain under three cases, respectively. For Γ_RAD_, the end of the test fixture is surrounded by commercial polyurethane foam absorber. For Γ_PEC_, the end of the parallel-plate waveguide is covered by a metallic plate with the same dimensions as the absorber sample. For Γ_DUT_, the absorber sample is placed near the end of the test fixture. According to equation (1), we can obtain the absorptivity. This method is very efficient for long-wavelength band (on the orders of meters) measurement, where free space measurement cannot be conveniently and inexpensively implemented.

## Electronic supplementary material

Supplementary Information
