# Supplementary material for: Broadband and thin magnetic absorber with non-Foster metasurface for admittance matching
Source: Sci Rep. 2017 Jul 31;7:6922. doi: 10.1038/s41598-017-07323-4 (PMC5537361; doi:10.1038/s41598-017-07323-4)
Supplement: Supplementary file 1 — Supplementary Information [file 41598_2017_7323_MOESM1_ESM.docx]

Broadband and thin magnetic absorber with non-Foster metasurface for admittance matching

**Jinchao Mou and Zhongxiang Shen***

School of Electrical and Electronic Engineering, Nanyang Technological University, 50 Nanyang Avenue, 639798, Singapore. Correspondence and requests for materials should be addressed to Z. Shen (email: ezxshen@ntu.edu.sg)

**Supplementary Information**

**Property of the magnetic material.** The permittivity and permeability of the magnetic material is provided by ARC. Inc, as graphically summarized in Fig. S1.





**Figure S 1 Property of the magnetic material.** *ε_r_* (left axis) and *μ_r_* (right axis).

**Investigation on the Case of *G_S_*>*Y_0_*.** If the thickness of the magnetic material is reduced to 1 mm, then *G_S_* will be larger than *Y_0_*, as plotted in Fig. S2 (a). In this case, the NFMS should provide negative surface conductance (Fig. S2(a)) and inductance (Fig. S2(b)) to facilitate admittance matching. Fig. S2(c) compares the absorptivity before and after matching.


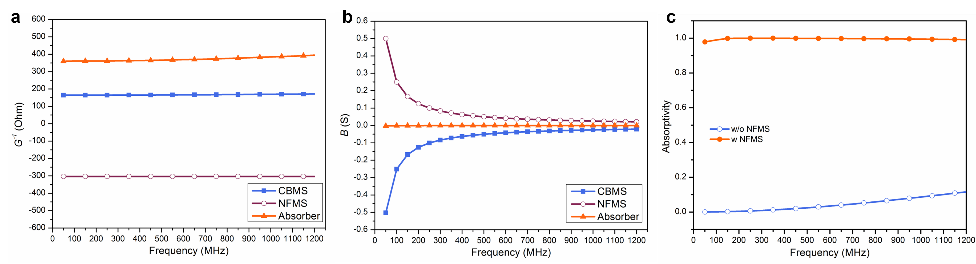


**Figure S 2 Simulated results of the case *G_S_*>*Y_0_*.** (a,b) *G* and *B* of the CBMS, NFMS, and the absorber. (c) Absorptivity of the CBMS without and with NFMS (absorber). The simulation is conducted based on the method used for Fig. 2.
